# Targeting HLA‐E in Lung Cancer: The Therapeutic Potential of IRF5‐Engineered M1‐Macrophage‐Derived Exosomes

**DOI:** 10.1111/crj.70035

**Published:** 2024-12-02

**Authors:** Xuqin Feng, Xiangyu Lai, Mingming Zhou, Jun Bie, Tingting Li, Dan Wang, Silin Chen, Xin Hu, Chunyu Wang, Peng Xu

**Affiliations:** ^1^ Department of Oncology Beijing Anzhen Nanchong Hospital, Capital Medical University (Nanchong Central Hospital), The Second Clinical Medical College of North Sichuan Medical College Nanchong Sichuan China; ^2^ Chongqing Key Laboratory of Translational Research for Cancer Metastasis and Individualized Treatment, Chongqing University Cancer Hospital, Chongqing Cancer Hospital Chongqing Cancer Institute Chongqing China; ^3^ Department of Clinical Laboratory Beibei Traditional Chinese Medical Hospital Chongqing China

**Keywords:** exosome, HLA‐E, lung cancer, macrophage, transcription factor

## Abstract

Immunotherapy is a pivotal approach in the treatment of lung cancer. Although HLA‐E is a potential target for tumor immunotherapy, its role in lung cancer remains unclear. Previous studies have identified the transcription factor IRF5 as a characteristic gene of M1‐like macrophages, highlighting its crucial role in promoting antitumor immune responses. In this study, we developed an engineered M1‐like macrophage exosomes expressing IRF5 (IRF5 M1‐exos) and demonstrated their ability to inhibit proliferation, migration, and invasion of lung cancer cells. Moreover, our experiments using a nude mouse model revealed that IRF5 M1‐exos exerted potent therapeutic effects by effectively suppressing tumor growth. Notably, the mechanism by which IRF5 exerts its antitumor function through HLA‐E regulation in lung cancer has not been fully elucidated. Here, we identified HLA‐E as a downstream target gene of IRF5 and demonstrated that the overexpression of HLA‐E can counteract the tumor‐promoting effects induced by si‐IRF5 M1‐exos. These results suggest that M1 macrophage‐derived exosomes, enriched with the transcription factor IRF5, exhibit potent antitumor activity by up‐regulating HLA‐E in lung cancer cells. Therefore, IRF5 M1‐exos represent an attractive therapeutic strategy for lung cancer.

## Graphical abstract

Schematic illustration of IRF5 M1‐exos: (A) shows the schematic presentation showing the preparation process of IRF5 M1‐exos; (B) shows the scheme showing the effects of IRF5 M1‐exos against cancer; and (C) shows the mechanism of anticancer effect of IRF5 M1‐exos.

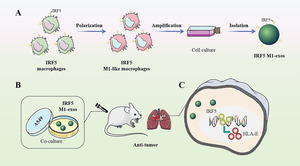

**Abbreviations**
HLA‐E: human leukocyte antigen‐E
TME: tumor microenvironment
TAMs: tumor‐associated macrophages
IRF5: interferon regulatory factor 5
LPS: lipopolysaccharide
ChIP: chromatin immunoprecipitation

## 1 Introduction

Lung cancer has the highest incidence and mortality rate globally, with approximately 1.8 million deaths per year and a 5‐year survival rate of less than 18% [1]. In 2020, an estimated 2.2 million new cases of lung cancer were diagnosed, and approximately 1.8 million deaths were attributed to this disease. Lung cancer is the leading cause of cancer‐related deaths in men worldwide, ranking second among women (after breast cancer) [2]. Conventional treatments for lung cancer, including surgical intervention, chemotherapy administration, and radiation therapy, have been widely utilized. Despite significant progress in early detection and management of advanced lung cancer, the prognosis for patients remains poor. This can be primarily attributed to the complex biological characteristics and extensive heterogeneity of the tumor, along with its resistance to conventional chemoradiotherapy [3]. Following chemotherapy and molecular targeted therapy, immunotherapy has emerged as a prominent approach in the treatment of lung cancer and is now considered a standard therapeutic option for patients with this condition [4]. However, the outcomes of immunotherapy clinical trials are not consistently positive. A comprehensive understanding of the mechanism underlying immunotherapy treatment is crucial for enhancing the therapeutic efficacy and improving the quality of life for patients with lung cancer.

HLA‐E (human leukocyte antigen‐E) is considered a promising target for tumor immunotherapy. HLA‐E is a nonclassical MHC (major histocompatibility complex) Class I molecule that plays a crucial role in immune system regulation. In the field of cancer immunotherapy, HLA‐E exhibits high expression levels in various tumors, and its expression level correlates with the prognosis of tumor patients, depending on the tumor type [5, 6]. By binding to receptors on NK cells or T cells, HLA‐E may facilitate the evasion of detection and clearance by the immune system for tumor cells [7]. Liu et al. [8] discovered that HLA‐E plays a pivotal role in enabling circulating tumor cells to evade host immune surveillance, whereas circulating tumor cells (CTCs) avoid NK cell surveillance and inhibit tumor metastasis through an immune checkpoint molecular pair known as HLA‐E: CD94‐NKG2A. In addition to facilitating immune evasion, HLA‐E also exerts regulatory control over tumor growth through alternative mechanisms [9]. Nevertheless, the expression and role of HLA‐E in lung cancer remain elusive.

Immunotherapy has revolutionized cancer treatment by harnessing the power of the immune system to target and eliminate cancer cells. Within this therapeutic paradigm, macrophages have emerged as central players due to their dual role in shaping the tumor microenvironment (TME) [10]. Macrophages can be categorized into two distinct groups based on their phenotype and function: M1 type, characterized by its antitumor properties, and M2 type, known for promoting tumor progression [11]. M1 macrophages are known for their robust inflammatory response and ability to induce a Th1‐type immune response, making them potential effectors in cancer immunotherapy [12]. The M1 phenotype of tumor‐associated macrophages (TAMs) predominates in the early stages of tumorigenesis. However, as the tumor progresses, alterations in the TME gradually prompted a shift in TAM polarization from M1 to M2 phenotype [13]. Consequently, a predominant population of TAMs with an M2 phenotype is observed across most tumors. This suggests that enhancing the immune microenvironment surrounding tumors and transforming M2‐like TAMs into M1‐like macrophages can be considered as a viable approach to stimulate innate immunity against cancer [13].

The transcription factor IRF5 (Interferon regulatory factor 5), a crucial component of the interferon signaling pathway, plays a significant role in regulating immune responses and antiviral defense [14, 15]. Recent studies have revealed that IRF5 also contributes to tumor occurrence and development by regulating tumor immune escape and promoting antitumor immune response [16]. As a characteristic gene of M1‐type macrophages, IRF5 has been observed to promote polarization towards M1‐type in vitro, affecting the function of M1‐type macrophages and the survival of tumor cells [17]. Feng et al. [18] reported decreased expression of IRF5 in lung cancer patient tissues. M1‐like macrophages have the capability to secrete exosomes, facilitating the delivery of bioactive molecules to tumor cells and thereby exerting an indirect role in tumor therapy [19, 20]. Therefore, increasing the content of IRF5 in M1 macrophage exosomes may enhance its antitumor effect, offering a potential future treatment for lung cancer.

Despite limited research on the correlation between IRF5 and HLA‐E in human lung cancer, it is hypothesized that IRF5 could play a crucial role in regulating HLA‐E expression. In this study, we generated M1‐like macrophage exosomes overexpressing IRF5, referred to as IRF5 M1‐exos, for potential cotherapy in cancer treatment. To achieve this, we employed gene editing techniques to construct macrophages with enhanced IRF5 expression (IRF5 macrophages). Subsequently, these IRF5 macrophages were selectively induced into M1‐like phenotype, and their exosomes, containing high levels of IRF5, were collected. The effect of IRF5 M1‐exos on the proliferation and metastasis of lung cancer cells were observed through in vitro coculturing with lung cancer cells. In vivo, the inhibitory effect of IRF5 M1‐exos on tumor growth was observed by establishing a tumor‐bearing model in nude mice. We investigated the impact of IRF5 M1‐exos on lung cancer cell proliferation and tumor growth and, for the first time, identified that alterations in IRF5 expression result in significant upregulation of HLA‐E expression and promoter activity in lung cancer cell lines. Furthermore, we validated the role of HLA‐E in regulating lung cancer growth mediated by IRF5 M1‐exos. These findings indicated that exosomal transcription factor IRF5 derived from M1‐like macrophages plays an antitumor role by inducing upregulation of HLA‐E expression in lung cancer cells.

## 2 Materials and Methods

### 2.1 Cell Culture

Human lung cancer cells (A549 and SPC‐A1) were cultured in Dulbecco's Modified Eagle Medium (DMEM) containing 10% fetal bovine serum, penicillin (100 units/mL), and streptomycin (100 μg/mL). The RAW 264.7 murine macrophage cell line (ATCC®TIB‐71, Manassas, Virginia, United States) was maintained in Roswell Park Memorial Institute 1640 medium (RPMI 1640, Corning, Manassas, Virginia, United States). The HEK293T cells were grown in Dulbecco's modified Eagle's medium (Gibco) supplemented with 5% fetal bovine serum (FBS). All cells were cultured in a CO_2_ incubator at 37°C.

### 2.2 Lentiviral Transduction

IRF5 lentivirus was used to establish a stable population of IRF5‐expressing macrophages, known as IRF5 macrophages, in RAW 264.7 cells. To achieve this, RAW 264.7 cells were infected with IRF5 lentivirus at a multiplicity of infection (MOI) of 10, and the infectivity of the lentivirus was enhanced by adding polybrene. Following infection, puromycin selection (2–4 μg/mL) was applied to isolate cells that consistently expressed IRF5. Subsequently, the maintenance of IRF5 expression in the cultured IRF5 macrophages was ensured by supplementing the medium with puromycin (1–2 μg/mL). The lentiviral packaging system was used to perform HLA‐E overexpression in A549 and SPC‐A1 cells, which were infected to overexpress HLA‐E using the HLA‐E‐specific lentiviral vector (Shanghai Genechem Co., Ltd, Shanghai, China).

### 2.3 Directed Induction Into M1‐Like Macrophages

IRF5 M1‐like macrophages were generated by stimulating cultured IRF5 macrophages with lipopolysaccharide (LPS) at concentrations ranging from 100 to 500 ng/mL for durations of 24 to 48 h. The expression of IRF5 and the M1‐like macrophage marker CD86 were detected using immunofluorescence to analyze the IRF5 M1‐like macrophages. Similarly, M1‐like macrophages were obtained by culturing RAW 264.7 cells using the same method.

### 2.4 qRT‐PCR

The Rneasy Mini Kit (Qiagen, Austin, TX) was utilized for the extraction of total RNA. To synthesize cDNA, the Prime Script RT Reagent Kit (Takara, Dalian, China) was employed. An equal amount of cDNA was combined with SYBR green master mix (Applied Biosystems, United States), followed by performing qPCR on the 7500 real‐time PCR system (Applied Biosystems). The comparative threshold cycle delta Ct equation was used to calculate gene relative expression. For normalization purposes, GAPDH served as a housekeeping gene. Primer sequences: 5′‐ACCACAGTCCATGCCATCAC‐3′ (forward) and 5′‐TCCACCACCCTGTTGCTGTA‐3′ (reverse) for GAPDH. 5′‐TGTGCCAGTGTAAGGTGTTC‐3′ (forward) and 5′‐TTGAGAAACTGCTCTAGGCTAAAG‐3′ (reverse) for IRF5. 5′‐CACGTGCCATGTGCAGCA‐3′ (forward) and 5′‐CACAGCTCCAGAGACCA‐3′ (reverse) for HLA‐E.

### 2.5 Western Blot Analysis

Lysis was conducted in cells using RIPA buffer (manufactured by Thermo Scientific, United States) for the extraction of total protein. After performing SDS‐PAGE gel electrophoresis and transferring the proteins onto PVDF membranes, the membranes were blocked with skim milk. They were then incubated overnight with specific primary antibodies at a dilution ratio of 1:2000. Subsequently, HRP‐linked secondary antibody was applied for 1 h before the membranes were subjected to enhanced chemiluminescence (ECL) substrate to visualize the bands. All antibodies used in this study, including anti‐IRF5, anti‐CD63, anti‐Calnexin, anti‐HLA‐E, and anti‐GAPDH, were purchased from Cell Signaling Technology (Danvers, MA, United States).

### 2.6 Preparation of IRF5 M1‐Exos

IRF5 M1‐like macrophages were incubated in DMEM containing 10% exosome‐free FBS, and the supernatant from the cell culture was harvested. Subsequently, a series of centrifugation steps at different speeds (300×*g* for 10 min, followed by 2000×*g* for 10 min, and then 10 000×*g* for 30 min) were performed to eliminate cells, cell fragments, and smaller debris. The resulting supernatant was further subjected to ultracentrifugation at 100 000×*g* for 2 h. Afterwards, it was washed twice with PBS to isolate IRF5 M1‐exos. Similarly, M1‐exos were obtained from M1‐like macrophages using the same method. Finally, these isolated exosomes were suspended in PBS and stored at −80°C until further use.

### 2.7 Transmission Electron Microscopy

Transmission electron microscopy was used to observe the morphology of IRF5 M1‐exos. After the exosomes were absorbed onto carbon‐coated copper grids, the IRF5 M1‐exos suspension was subjected to negative staining with a solution of uranyl acetate, followed by rinsing with distilled deionized water. Subsequently, the grids were air dried prior to the detection and imaging of exosomes using TEM.

### 2.8 Viability Assay

In brief, A549 and SPC‐A1 cells were enzymatically digested, followed by centrifugation and subsequent seeding into 96‐well plates for routine culture. Then, 10 μL of CCK‐8 solution (Beyotime, Shanghai, China) was added to each well at the appropriate time point. The plate was then incubated at 37°C for 2 h in a controlled environment. Subsequently, the optical density at 450 nm (OD_450_) was measured using an enzyme‐linked immunosorbent assay reader.

### 2.9 Proliferation Detection

To assess cell proliferation, we utilized a Cell‐Light EdU DNA Cell Proliferation Kit (Ribobio, Guangzhou, China). A549 and SPC‐A1 cells were seeded at a density of 2.5 × 10^4^ in 96‐well plates and treated with EdU for 2 h at a concentration of 50 μM. Following fixation with paraformaldehyde (4%, 30 min) and permeabilization with Triton X‐100 (0.5%, 10 min), the cells were incubated with Apollo solution (1×, 30 min) and then stained with DAPI. After staining, the cells were visualized using fluorescence microscopy. The number of positive cells was counted and expressed as a ratio of the total number of cells.

### 2.10 Colony Formation Ability

The cells were incubated in six well plates at 37°C, with a density of 1 × 10^3^ cells per well. After a 2‐week period, the colonies were fixed with methanol and subjected to crystal violet staining. Subsequently, the plates were scanned for colony enumeration.

### 2.11 Wound Healing Assay

Lung cancer cells were evenly distributed onto six‐well plates and cultured in medium containing FBS until they reached 90% confluence. A sterile pipette tip was used to swiftly create a straight scratch across the cell monolayer. Subsequently, serum‐free medium was added to the plates, and the cells were allowed to culture for 24 h. The migration process was captured using an Olympus CKX41 inverted microscope (Tokyo, Japan) and analyzed using ImageJ software (NIH, Bethesda, MD, United States). To determine the migration rate (MR), we employed the following formula: *MR* = (*A* − *B*)/*A* × 100%, where *A* represents the initial width at 0 h and *B* represents the width after 24 h of culture.

### 2.12 Transwell Assay

For the invasion assay, the upper membrane of transwell chambers (Corning, United States) was precoated with a mixture of matrigel matrix (1:6, BD Science, United States). In contrast, for the migration assay, no matrigel matrix was used. To perform the assays, we added a cell suspension consisting of 2 × 10^4^ cells in 100‐μL DMEM medium to the upper chamber. Subsequently, we added 700 μL of DMEM medium containing 10% serum to the lower chamber. After a 24‐h incubation, the cells on the lower surface were fixed and stained using paraformaldehyde (4%) and crystal violet dye (0.1%). Finally, the stained cells were counted.

### 2.13 Animal Model

BALB/c nude mice (male, 5 weeks old) with similar weight were utilized for in vivo analysis. All animal procedures were approved by the Medical Ethics and Welfare Committee for Experimental Animals of Nanchong Central Hospital. After 1 week of acclimation under SPF standard conditions, lung cancer xenografts were established in nude mice. A549 cells (2 × 10^6^), cocultured with M1‐exos or IRF5 M1‐exos, were resuspended in 200 μL of PBS and subsequently injected into the right axilla of the nude mice. After a 28‐day duration, the nude mice were euthanized, and tumor tissue was excised and weighed.

### 2.14 Bioinformatics Analysis

GEPIA is a comprehensive cancer genomics dataset that integrates extensive data from The Cancer Genome Atlas (TCGA) and the Genotype‐Tissue Expression project (GTEx) (http://gepia.cancer‐pku.cn/). It was utilized to analyze gene expression variations between LUAD and normal tissues via analysis of variance (ANOVA), generating box plots. Additionally, GEPIA was used to assess the correlation between IRF5 and HLA‐E, employing the Pearson correlation coefficient as the statistical method.

The Gene Expression Omnibus (GEO) dataset, The HUMAN PROTEIN ATLAS database (https://www.proteinatlas.org/), and UALCAN database (https://ualcan.path.uab.edu/) were utilized for the analysis of HLA‐E expression in lung cancer tissues. The Kaplan–Meier database was employed to investigate the correlation between HLA‐E expression and survival rates among lung cancer patients.

The Tumor Immune Estimation Resource (TIMER) database was used to explore the association of HLA‐E expression with immune infiltration. The National Center for Biotechnology Information (NCBI) database (https://www.ncbi.nlm.nih.gov/) was utilized to retrieve the HLA‐E promoter sequences. Jaspar, a database of eukaryotic transcription factors (http://jaspar.genereg.net/), was employed in conjunction with the spectral database for obtaining the DNA sequence of IRF5, its identification figure, and predicting potential binding sites of IRF5 and HLA‐E within the DNA promoter.

### 2.15 Chromatin Immunoprecipitation (ChIP) and Sequencing

The ChIP assay was conducted in accordance with the manufacturer's instructions (Abcam, Cambridge, MA, United States) using the A549 and SPC‐A1 cell lines. Protein A/G beads were combined with an equal amount of anti‐IRF5‐1 antibody or rabbit IgG (Proteintech) and incubated overnight at 4°C. The A549 and SPC‐A1 cells were fixed using 3.7% formaldehyde. After 10 min, glycine (125 mM) was added to halt the cross‐linking reaction caused by formaldehyde. The cells were then collected into an Eppendorf (EP) tube and centrifuged at 1000×*g* for 10 min at 4°C. The supernatant was removed, and the pellet was resuspended in cytoplasmic lysis buffer (5‐mM PIPES pH 8.0, 85 mM KCl, 0.5% Nonidet P‐40 containing protease inhibitors; volume: 1000 μL per every one million cells). Subsequently, the cell suspension underwent centrifugation at a speed of 4000×*g* for 5 min at a temperature of 4°C. The resulting pellet was resuspended in nuclear lysis buffer (50‐mM Tris–HCl pH 8 0.1, 10‐mM EDTA,1% SDS supplemented with protease inhibitors; volume: 500 μL per every one million cells), followed by sonication for 10 cycles consisting of alternating periods of 30 s on and 30 s off for each cycle. On the following day, the DNA‐protein complex was mixed with the antibody‐A/G beads complex and allowed to incubate at a temperature of 4°C for 10 h. Subsequently, the chromosomal DNA underwent purification before being subjected to quantitative PCR analysis or sent out for high‐throughput sequencing (Igenebook, Wuhan). Finally, the data obtained from these experiments were analyzed by LC Biotech.

### 2.16 Luciferase Reporter Assay

Prior to transfection, 293‐T cells were seeded at a density of 2 × 10^5^ cells per well in a six‐well cell culture plate. Each DNA construct (2 μg) and the Renilla construct (0.3 μg, Promega) were combined with lipo3000 (10 μL, Qiagen, Germany). At room temperature, the mixture was incubated for 20 min. Following washing with PBS, the DNA/lipo3000 mixtures were added to the cells and cultured in a CO_2_ incubator at 37°C for 6 h. The complete culture medium replaced the supernatant. When cotransfecting the constructs of individual transcription factors, separately cultured cells were transfected with appropriate control plasmids pGL3 as controls. The DNA content was maintained equally throughout the experiment. Subsequently, the transfected cells underwent PBS washes and continued cultivation for an additional 48 h. After this period, reporter lysis buffer (Promega) was used to lyse the transfected cells. Firefly and Renilla luciferase enzymatic activities were measured using the Dual‐Luciferase Reporter assay System (Promega) and a luminometer. All luciferase assays were conducted in duplicate or triplicate, and each experiment was replicated at least twice.

### 2.17 Statistics

All data were represented as mean ± standard error of the mean. Student's *t* test or one‐way analysis of variance (ANOVA) followed by Tukey's test was performed using GraphPad Prism8 software to compare the difference. The survival was analyzed with Kaplan–Meier method and log‐rank test. *p* < 0.05 is regarded as statistically significant.

## 3 Results

### 3.1 Preparation and Characterization of IRF5 M1‐Exos

**FIGURE 1:**
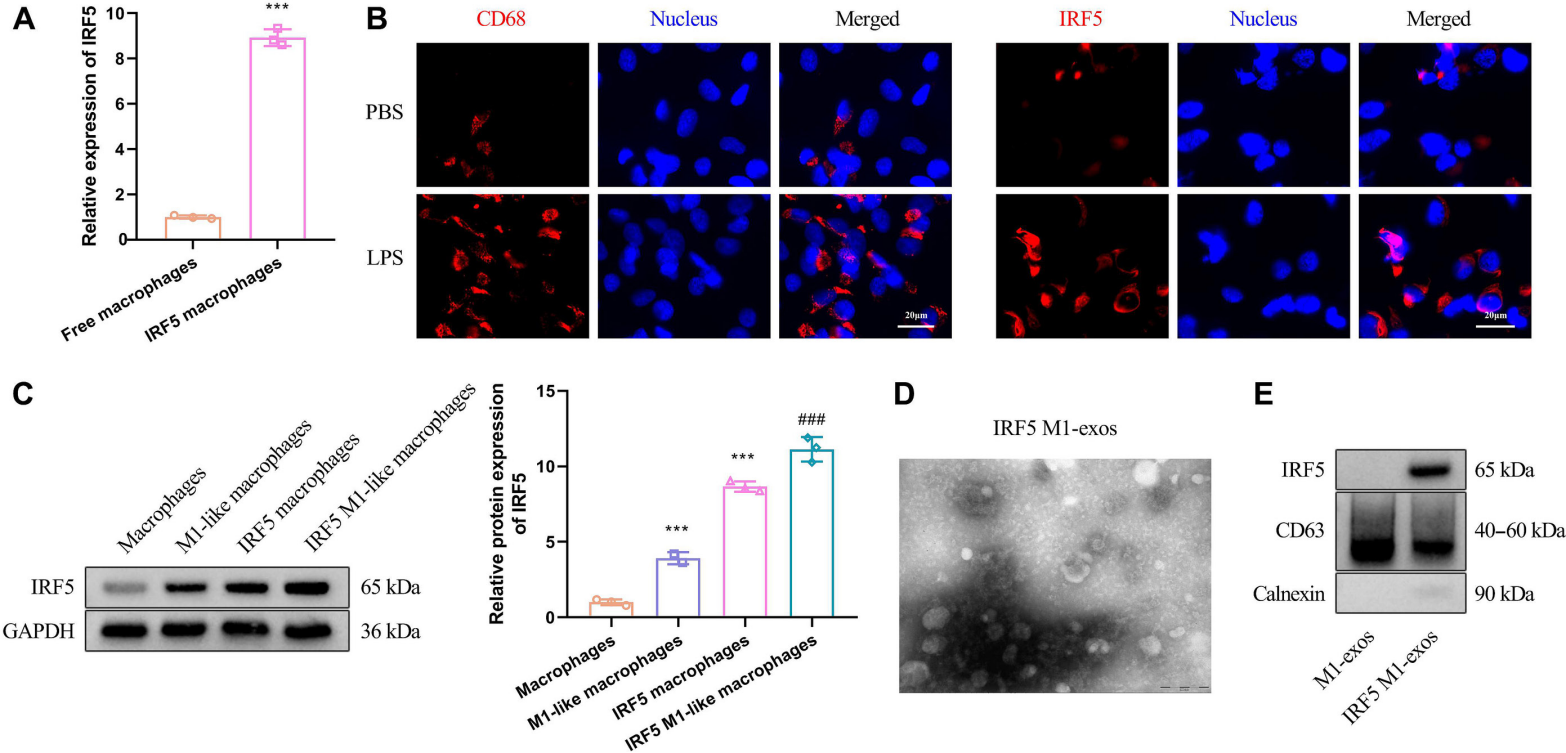
Preparation and characterization of IRF5 M1‐exos. (A) RT‐qPCR detection of IRF5 mRNA levels in macrophages and IRF5 macrophages. (B) Confocal images showing CD86 (M1‐like macrophage marker) and IRF5 expression in IRF5 M1‐like macrophages. Scale bar, 20 μm. (C) Western blot analysis of IRF5 protein levels in macrophages and IRF5 macrophages. (D) Transmission electron microscope (TEM) image of IRF5 M1‐exos. Scale bar, 200 nm. (E) Western blot analysis of the protein levels of IRF5 and CD63. Compared with free macrophages, ***p < 0.001; compared with macrophages, ***p < 0.001; compared with M1‐like macrophages, ^###^ *p* < 0.001.

Macrophages overexpressing IRF5 were obtained through lentivirus infection. Real‐time quantitative polymerase chain reaction showed that IRF5 mRNA levels in IRF5 macrophages were significantly higher than those in free macrophages (Figure 1A). The directional polarization of IRF5 macrophages into IRF5 M1‐like macrophages was induced by LPS. Immunofluorescence analysis demonstrated that the polarization induced by LPS led to a notable increase in CD86 expression within IRF5 macrophages, in which IRF5 remained significantly overexpressed (Figure 1B). Western blot analysis of IRF5 protein expression in macrophages showed that IRF5 was significantly overexpressed in IRF5 macrophages. The protein content of IRF5 was higher in IRF5 M1 macrophages than in IRF5 macrophages (Figure 1C). These results indicated that we have successfully obtained IRF5 M1‐like macrophages.

IRF5 M1‐like macrophages were cultured, and the supernatant was collected. IRF5 M1‐exos were isolated from the supernatant through differential centrifugation. As depicted in Figure 1D, the morphology and structure of IRF5 M1‐exos were examined using nanoparticle tracking analysis and transmission electron microscopy imaging. The results demonstrated that the exosomes exhibited a hemispherical or saucer‐like shape with one side concave, whereas their particle size ranged from 50 to 150 nm. Western blot analysis revealed a significant upregulation of IRF5 in IRF5 M1‐exos compared to M1‐exos (Figure 1E). Collectively, these experimental findings demonstrate the successful preparation of IRF5 M1‐exos.

### 3.2 IRF5 M1‐Exos Inhibits Lung Cancer Cell Proliferation, Metastasis, and Tumor Growth

**FIGURE 2:**
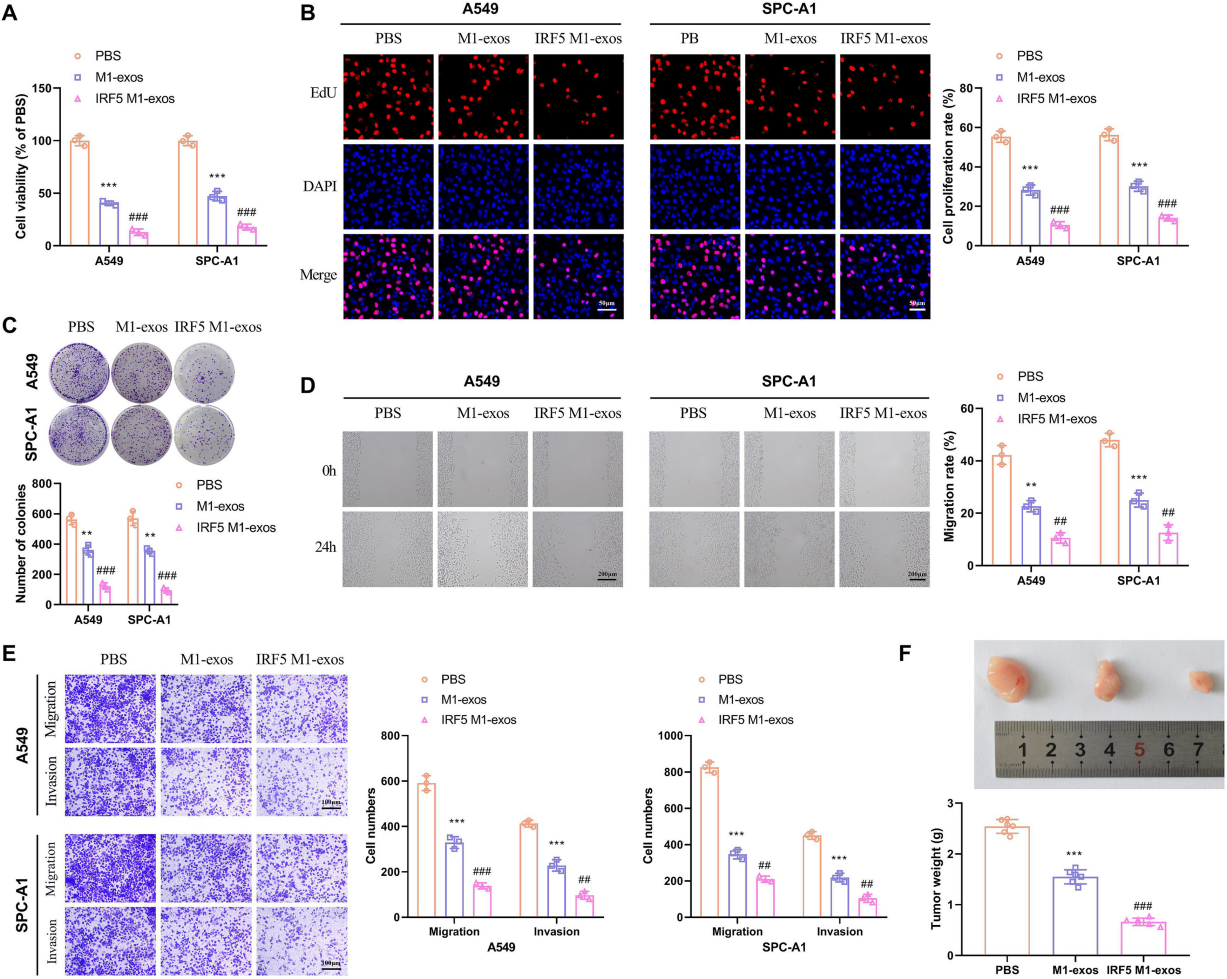
IRF5 M1‐exos inhibits lung cancer cell proliferation, metastasis and tumor growth. (A) The percentages of viable cells were determined using CCK‐8 and the cell viability of the PBS group was taken as 100%. (B) DNA replication activities of A549 and SPC‐A1 cells in each group were examined by EdU assay; original magnification 100X. (C) The clonogenic capacity was determined in A549 and SPC‐A1 cells. (D) The effect of IRF5 M1‐exos on cell migration was measured by wound healing assay; original magnification 100X. (E) Transwell assay was performed to assess the migration and invasion of A549 and SPC‐A1 cells; original magnification 200X. (F) Representative xenografts dissected from different groups of nude mice were shown, and the tumors were weighed. Compared with PBS, **p < 0.01, ***p < 0.001; compared with M1‐exos, ^##^ *p* < 0.01, ^###^ < 0.001.

To elucidate the impact of IRF5 M1‐exos on lung cancer cells, we conducted coculture experiments with the A549 or SPC‐A1 cell lines using PBS, M1‐exos, and IRF5 M1‐exos. The Cell Counting Kit‐8 (CCK‐8) assay revealed that M1‐exos diminished the metabolic activity and viability of A549 and SPC‐A1 cells, with IRF5 M1‐exos exerting a more pronounced effect (Figure 2A). As expected, the cell proliferative capacity was significantly attenuated when cocultured with either M1‐exos or IRF5 M1‐exos (Figure 2B). Furthermore, M1‐exos decreased the ability of cells to form colonies, and IRF5 M1‐exos augmented this effect (Figure 2C). Subsequently, a wound healing assay indicated that cells in the IRF5 M1‐exos group migrated slower than that in the PBS group, followed by the M1‐exos group (Figure 2D). Transwell assay results revealed fewer migrated and invaded cells in the M1‐exos and IRF5 M1‐exos groups (Figure 2E). Additionally, to investigate the impact of IRF5 M1‐exos on lung cancer tumor growth, we inoculated A549 cells cocultured with M1‐exos or IRF5 M1‐exos into nude mice and successfully established xenograft models. As shown in Figure 2F, both the M1‐exos and IRF5 M1‐exos groups exhibited significantly suppressed tumor growth rates compared to the PBS group, with the smallest tumor size observed in the IRF5 M1‐exos group (Figure 2F). Collectively, these findings conclusively demonstrate that IRF5 M1‐exos possess inhibitory effects on lung cancer cell survival and proliferation while attenuating cellular migration and invasion capabilities, thereby impeding tumorigenesis.

### 3.3 HLA‐E Is a Target Gene for the Transcription Factor IRF5

**FIGURE 3:**
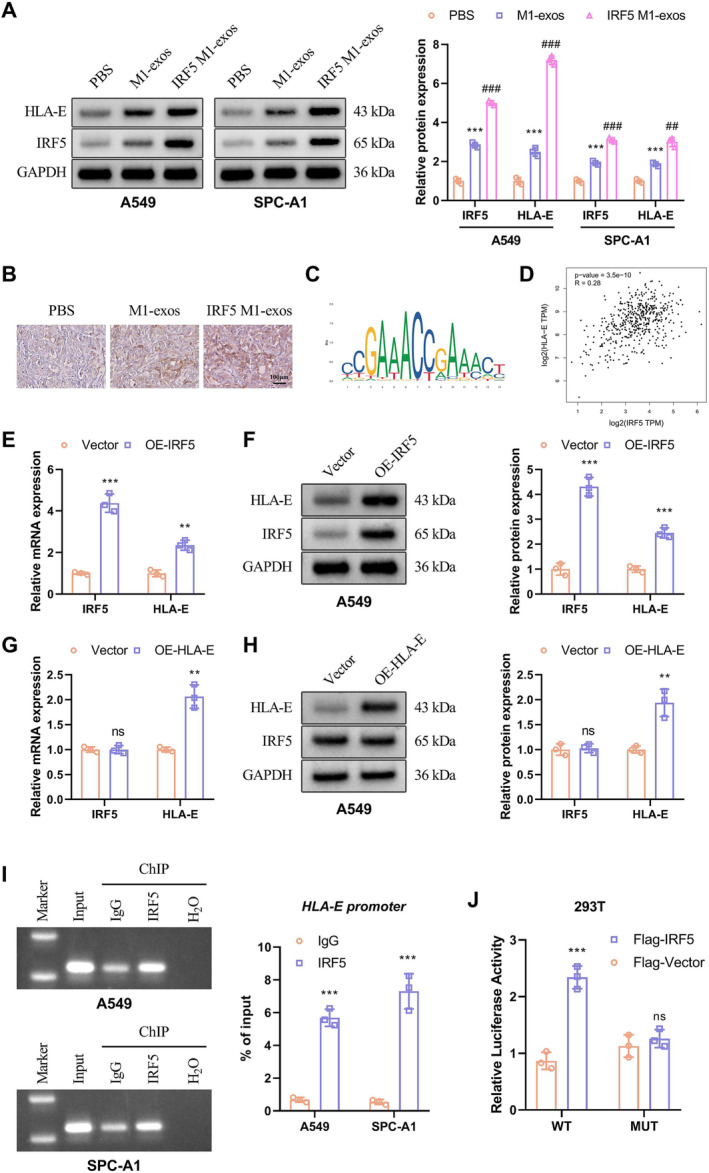
HLA‐E is a target gene for the transcription factor IRF5. (A) Western blot analysis was applied to measure protein expression levels of IRF5 and HLA‐E in A549 and SPC‐A1 cells cocultured with IRF5 M1‐exos. (B) Representive photographs of HLA‐E staining in tumor tissues. (C) The sequence logo representing DNA binding sites for IRF5. (D) The correlation of IRF5 and HLA‐E in LUAD (GEPIA2 analysis). (E) qRT‐PCR was performed to detect the mRNA levels of IRF5 and HLA‐E in OE‐IRF5‐transfected A549 cells. (F) Western blot was conducted to detect the protein levels of IRF5 and HLA‐E in OE‐IRF5‐transfected A549 cells. (G) qRT‐PCR was performed to detect the mRNA levels of IRF5 and HLA‐E in OE‐HLA‐E‐transfected A549 cells. (H) Western blot was conducted to detect the protein levels of IRF5 and HLA‐E in OE‐ HLA‐E‐transfected A549 cells. (I) Chromatin immunoprecipitation (ChIP) assay performed with IRF5 antibody followed by detection of *HLA‐E* promoter by RT‐qPCR. (J) After cotransfection of IRF5 and a luciferase reporter gene in HEK293T cells, the activity of HLA‐E is detected by luciferase assay. Compared with PBS, ***p < 0.001; compared with M1‐exos, ^##^ *p* < 0.01, ^###^ *p* < 0.001; compared with vector, **p < 0.01, ***p < 0.001; ns indicates a lack of statistically significant differentiation; compared with IgG, ***p < 0.001; compared with flag‐vector, ***p < 0.001; ns indicates a lack of statistically significant differentiation.

Next, we investigated the downstream regulatory mechanism of IRF5. Western blot analysis revealed an increase in HLA‐E protein expression in the M1‐exos group compared to the PBS group, and lung cancer cells cocultured with IRF5 M1‐exos exhibited higher levels of HLA‐E expression (Figure 3A). Furthermore, immunohistochemical experiments conducted on transplanted tumor models in nude mice confirmed significantly elevated levels of HLA‐E expression in tumor tissues from the IRF5 M1‐exos group compared to the M1‐exos group (Figure 3B). To explore potential correlations between IRF5 and HLA‐E, we utilized the JASPAR database of eukaryotic transcription factor binding profiles to predict three possible binding sites within the DNA promoters for both IRF5 and HLA‐E. Figure 3C displays the sequence logo representing these DNA binding sites for IRF5. Analysis using GEPIA database demonstrated a positive correlation observed between IRF5 and HLA‐E (R = 0.28, *p* < 0.001) (Figure 3D). To further investigate the mutual regulation of IRF5 and HLA‐E both in vivo and in vitro, lentivirus‐transfected A549 cells overexpressing either IRF5 or HLA‐E were generated. Interestingly, overexpression of IRF5 led to an increase in both protein and mRNA levels of HLA‐E in A549 cells; however, overexpression of HLA‐E had no effect on either protein or mRNA levels of IRF‐5 (Figure 3E–H). Next, we conducted ChIP experiments and observed that the IRF5 antibody specifically precipitated the predicted HLA‐E promoter fragment, whereas the control IgG did not show any specific binding (Figure 3I). Additionally, luciferase reporter assays demonstrated a significant increase in HLA‐E promoter activity upon overexpression of IRF5 (Figure 3J). These findings suggest that IRF5 may exert its transcriptional regulatory role by activating HLA‐E expression.

### 3.4 The Expression of HLA‐E Is Decreased in Lung Cancer Tissues

**FIGURE 4:**
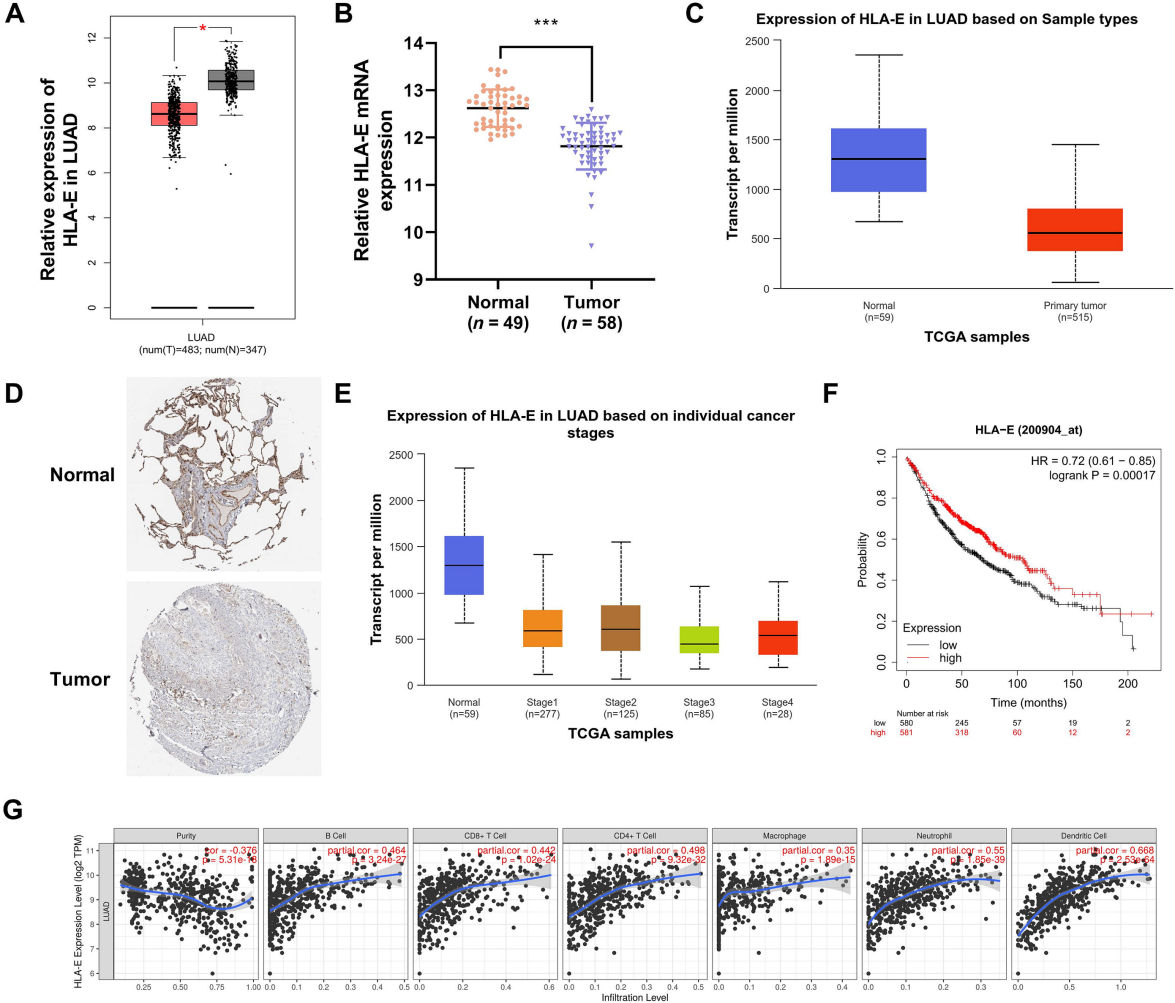
The expression of HLA‐E is decreased in lung cancer tissues. (A) The expression patterns of HLA‐E in LUAD and normal tissues (GEPIA2 analysis). (B) The expression patterns of HLA‐E in LUAD and normal tissues (GEO dataset). (C) HLA‐E expression levels in LUAD patient cohort from TCGA database were analyzed using online tool UALCAN. The dataset included 515 LUAD tumor tissues and 59 normal‐tissue samples. (D) Immunohistochemistry showed the expression of HLA‐E in LUAD and paracancerous tissues (The HUMAN PROTEIN ATLAS database). (E) UALCAN database indicated a correlation between HLA‐E expression levels and individual stages of lung cancer. (F) Kaplan–Meier curves showed that low expression of HLA‐E predicted the poorer overall survival (OS) of LUAD patients (Kaplan–Meier plotter database).

Given that HLA‐E is a downstream target gene of IRF5, we speculated that HLA‐E may be involved in the regulation of lung cancer cell growth by IRF5. Therefore, we initially examined the expression of HLA‐E in lung cancer tissues and explored its association with survival outcomes. The GEPIA database showed that HLA‐E expression levels in lung cancer tissues were significantly downregulated (347 normal‐tissue samples and 483 tumor samples) (Figure 4A), and the GEO dataset (49 normal‐tissue samples and 58 tumor samples, *p* < 0.0001) (Figure 4B) and TCGA datasets (59 normal‐tissue samples and 515 tumor samples, *p* < 0.0001) (Figure 4C) also showed similar results, with significantly reduced HLA‐E mRNA levels in lung cancer tissues. Furthermore, immunohistochemical information of HLA‐E was retrieved from The HUMAN PROTEIN ATLAS database. It was found that antibody CAB024589 was used for immunohistochemical analysis of both cancer and normal tissues, revealing a significantly elevated expression of HLA‐E protein in lung cancer tissues compared to adjacent normal tissues (Figure 4D). Additionally, the UALCAN database indicated a correlation between HLA‐E expression levels and individual stages of lung cancer (Figure 4E). Kaplan–Meier analysis further revealed reduced survival rates among patients with low HLA‐E expression (*p* = 0.00017) (Figure 4F). Results from the TIMER database revealed a significant positive correlation between HLA‐E expression and the expression of CD8^+^ T cells, CD4^+^ T cells, macrophages, neutrophils, and dendritic cells in LUAD (Figure 4G). Combining the above results, we speculate that HLA‐E may be related to the occurrence and development of lung cancer. In addition, HLA‐E is likely to modulate the tumor immune microenvironment, thereby impacting the survival rates of patients with LUAD.

### 3.5 Overexpression of HLA‐E Can Reverse the Tumor‐Promoting Effect of si‐IRF5 M1‐Exos

**FIGURE 5:**
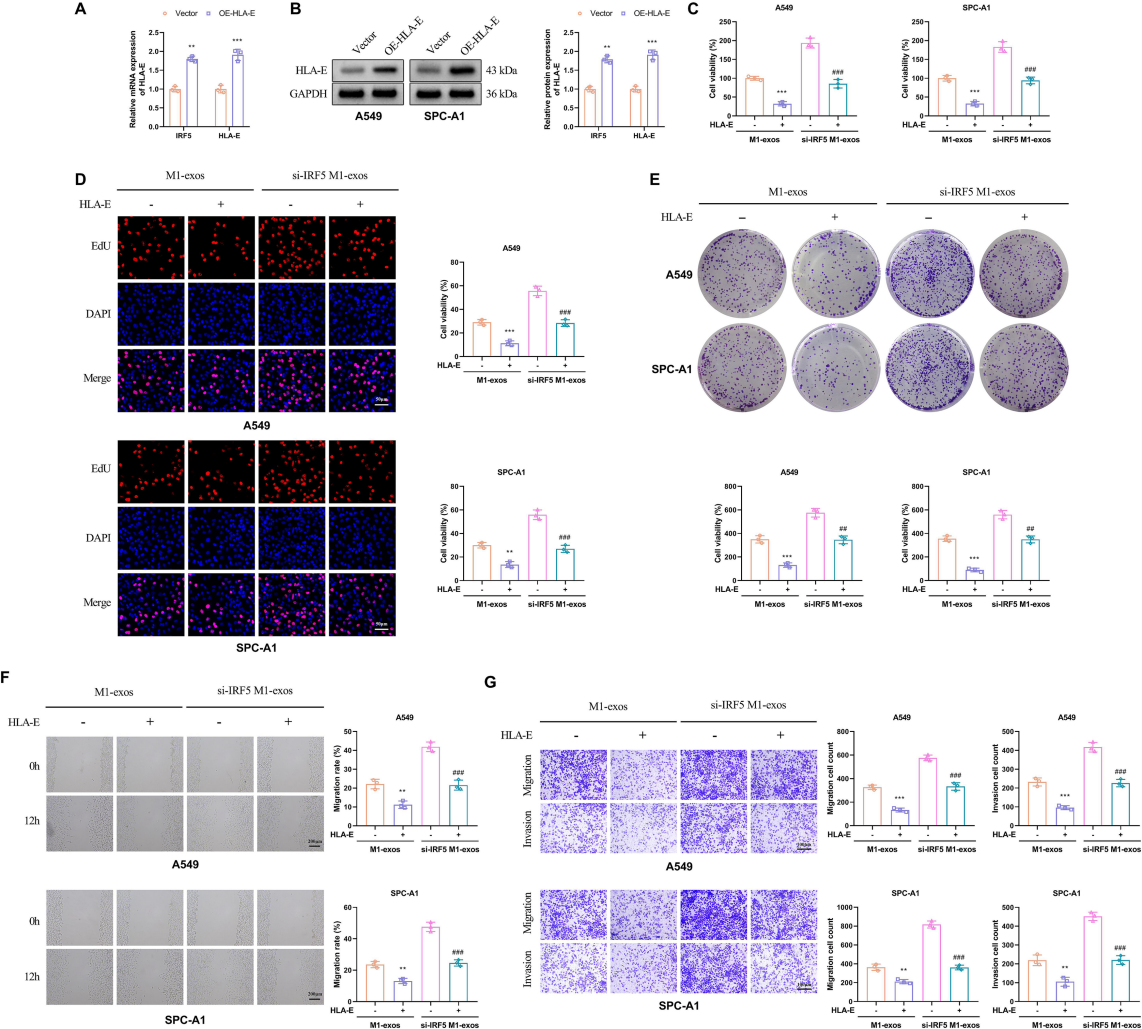
Overexpression of HLA‐E can reverse the tumor‐promoting effect of si‐IRF5 M1‐exos. (A) qRT‐PCR was performed to detect the mRNA levels of HLA‐E in A549 and SPC‐A1 cells. (B) The protein expression of HLA‐E was examined in A549 and SPC‐A1 cells by Western blot. GAPDH was used as the loading control. (C) CCK‐8 assay was used to detect the cell activity. (D) EdU assay was conducted to determine cell proliferation capacity; original magnification 100X. (E) Colony formation assay was performed to detect the clonogenic ability of A549 and SPC‐A1 cells. (F) Scratch assay was used to detect the wound healing ability of A549 and SPC‐A1 cells. (G) Transwell assay was utilized to detect the migration and invasion ability of A549 and SPC‐A1 cells. Compared with vector, **p < 0.01, ***p < 0.001; compared with M1‐exos+Vector, **p < 0.01, ***p < 0.001; compared with si‐IRF5 M1‐exos + vector, ^##^ *p* < 0.01, ^###^ *p* < 0.001.

Next, we further investigated the potential involvement of HLA‐E in the growth of lung cancer cells mediated by IRF5. Lentiviral vectors were employed to overexpress HLA‐E in A549 and SPC‐A1 cells. qRT‐PCR and Western blot analysis revealed a significant increase in both mRNA levels and protein content of HLA‐E in the OE‐HLA‐E group compared to the Vector group (Figure 5A,B). To generate IRF5‐deficient M1 macrophages, si‐RNA was used to knock down IRF5, followed by extraction of exosomes (si‐IRF5 M1‐exos). Coculturing M1‐exos or si‐IRF5 M1‐exos with lung cancer cells from both Vector and OE‐HLA‐E groups demonstrated that overexpression of HLA‐E led to decreased metabolic activity and viability of A549 and SPC‐A1 cells, irrespective of coculture conditions (Figure 5C). Furthermore, cell proliferation and colony‐forming ability were significantly reduced (Figure 5D,E), whereas cell migration and invasion capacity were inhibited (Figure 5F,G). Collectively, these findings suggest that IRF5 regulates lung cancer cell growth through its interaction with HLA‐E, wherein overexpression of HLA‐E can counteract the tumor‐promoting effects induced by si‐IRF5 M1‐exos.

## 4 Discussion

Cells belonging to the monocyte–macrophage lineage are crucial constituents of the inflammatory aspect within the ecological habitat of cancer and significantly impact its progression [21]. However, the effects of M1 and M2 macrophages in the body are opposite due to their distinct phenotypes. M1 macrophages have the ability to release proinflammatory cytokines, which can impede tumor growth. Conversely, M2 macrophages possess the capability to promote tumor growth and metastasis [22]. M1 macrophages, which can be activated by various stimuli such as LPS, IFN‐γ, or TNF‐α, demonstrate a strong ability to present antigens and engulf particles through complement‐mediated phagocytosis. This contributes to the initiation of an inflammatory response [23]. Their proinflammatory functions aid in the elimination of foreign substances and impede tumor development [23]. Yu et al. [24] highlighted that the potential of inducing M1‐type polarization in macrophages and reversing the immunosuppressive microenvironment as a means to enhance the clinical efficacy of conventional lung cancer treatments. Exosomes, which reflect the characteristics of their parent cells, are an important means of communication between nontumor cells and tumor cells in the microenvironment, playing an important role in various stages of tumor development, including angiogenesis, tumor‐related immune regulation, microenvironment reconstruction, invasion, and distant metastasis [25]. Previous studies have demonstrated that the utilization of exosomes derived from M1 macrophages can ameliorate the immunosuppressive TME by facilitating the repolarization of TAMs towards the M1 phenotype, thereby enhancing the efficacy of cancer immunotherapy [26, 27, 28]. Therefore, M1‐like macrophage‐derived exosomes have the potential to serve as innovative therapeutic strategies for cancer treatment.

In this study, we developed IRF5 M1‐exos, which are derived from M1‐like macrophages that overexpress the IRF5 protein. Our findings demonstrate that, similar to M1‐exos, IRF5 M1‐exos significantly impedes the survival and proliferation of lung cancer cells while attenuating their migratory and invasive capabilities. Furthermore, in mouse models of lung cancer, the administration of IRF5 M1‐exos effectively suppressed tumor growth, surpassing the anticancer effects observed with conventional M1‐exos. Collectively, our engineered IRF5 M1‐exos represents a promising strategy for tumor immunotherapy.

HLA‐E overexpression has been detected in various solid and hematological malignancies. Studies indicate a correlation between elevated HLA‐E expression levels and unfavorable clinical outcomes in different cancer types, such as glioblastoma, ovarian carcinoma, multiple myeloma, vulvar squamous cell carcinoma, and gastric cancer [29, 30, 31, 32, 33]. The overexpression of HLA‐E in tumor cells, TAMs, and dendritic cells has the potential to hinder the immune response against tumors by restricting NKG2A^+^CD8^+^lymphocytes from infiltrating the TME [34]. Similarly, Zhen et al. [35] reported a significantly higher expression rate of HLA‐E in colorectal cancer tissues compared to adjacent normal tissues, with patients exhibiting higher HLA‐E expression showing lower survival rates. Conversely, our study demonstrated a significant lower expression of HLA‐E in lung cancer and its association with reduced survival. Furthermore, we discovered that overexpressing HLA‐E can counteract the tumor‐promoting effects of IRF5‐deficient M1‐like macrophage exosomes. This finding further validates the mechanism by which IRF5 regulates lung cancer cell growth through HLA‐E and offers potential avenues for developing novel therapeutic strategies.

Although our study provides significant insights into the role of IRF5 and HLA‐E in lung cancer treatment, there are still certain limitations. Our research primarily relies on in vitro experiments and nude mouse models, which may not fully recapitulate the intricate physiological environment within the human body. Furthermore, although we have established a positive regulatory relationship between IRF5 and HLA‐E, further investigations are required to elucidate how IRF5 modulates HLA‐E expression at the transcriptional level and to determine the specific implications of this process in lung cancer development.

In conclusion, our study reveals the potential role of IRF5 derived from M1‐like macrophage exosomes in lung cancer treatment through the upregulation of HLA‐E expression, thus providing a novel theoretical foundation for future immunotherapy strategies. Further investigations are warranted to explore the therapeutic potential of IRF5 and HLA‐E in lung cancer treatment.

## Author Contributions

Xuqin Feng and Xiangyu Lai administrated the project, implemented the experiment, and wrote the draft. Mingming Zhou, Jun Bie and Tingting Li collected the data and performed the statistical analysis. Dan Wang and Silin Chen are responsible for making the chart. Xin Hu contributed to the methodology. Chunyu Wang and Peng Xu designed and supervised the study. All authors reviewed the manuscript.

## Conflicts of Interest

The authors declare no conflicts of interest.

## Data Availability

Data are available from the corresponding author upon reasonable request.
